# Quality of Life after Flap Reconstruction of the Distal Lower Extremity: Is There a Difference Between a Pedicled Suralis Flap and a Free Anterior Lateral Thigh Flap?

**DOI:** 10.1097/GOX.0000000000002114

**Published:** 2019-04-04

**Authors:** Karsten Schmidt, Michael Gregor Jakubietz, Fabian Gilbert, Franca Hausknecht, Rainer Heribert Meffert, Rafael Gregor Jakubietz

**Affiliations:** From the Department of Trauma, Hand, Plastic and Reconstructive Surgery, Julius-Maximilians-University of Wuerzburg, Wuerzburg, Germany.

## Abstract

**Background:**

Flap reconstruction of the distal lower extremity is challenging. Especially, the concept of perforator surgery has increased available surgical options. Although results are generally judged in terms of objective facts, patients-perceived quality of life has largely remained unexamined. The aim of the study was to compare quality of life after lower extremity reconstruction with pedicled and free flaps.

**Methods:**

Patients were evaluated retrospectively after reconstruction of defects of the distal lower extremity either with distally based adipofascial sural flap (pedicled reverse sural flap) or an anterior lateral thigh (ALT) flap. A specific questionnaire was developed to measure the patient’s quality of life, based on short form health survey-12, Dresden Body Image Score-35, Patient Health Questionnaire-4, and X-SMFA questionnaires with additional specific questions. Furthermore, results, secondary surgeries, and complications were analyzed.

**Results:**

Thirty-seven patients with reconstruction of lower limb defects treated with a pedicled reverse sural flap and 34 patients treated with an ALT flap were included in the study. There was no statistical significant difference in the overall satisfaction with the procedure in the long-term follow-up between both groups, but patients with ALT showed a higher satisfaction with the treatment in the initial postoperative period. Both groups demonstrated approximately similar results in the long term for self-acceptance and vitality.

**Conclusions:**

Although anatomic situation may dictate flap choice coverage with free flaps, a less-complicated flap is by no means regarded as an inferior treatment option in patient’s estimation. Despite the intuitive speculation that patients with more advanced reconstruction methods should have better function and subsequently higher quality of life, this assumption was clearly not supported by data in this study.

## INTRODUCTION

Soft-tissue reconstruction of posttraumatic lower limb defects in the last decades was enhanced by the introduction of perforator flaps and by the improvement of the microsurgical preparation techniques. These techniques enable the plastic surgeon to offer multiple variants of soft-tissue reconstruction to the patient. The wound closure ladder^1^ which was renamed reconstructive ladder postulated by Levin^2^ has been overcome by individually and patient-specific reconstruction techniques^3,4^ and has led to a higher variability in modern reconstructive surgery. Especially, the widespread acceptance of perforator-based flaps has led to customized reconstruction methods while significantly reducing donor site morbidity. Although the surgical progression was acknowledged by the specialists, it remains unknown if patients feel a benefit in quality of life (QoL) after complex reconstructive procedures as opposed to more basic procedures. The most important aspect for patients was stable closure of the wound. In the surgical literature, most studies focus on objective clinical data such as donor site morbidity, blood loss, time of surgery, survival rate, etc., whereas patient perception in the long term as the ultimate evaluation of surgical procedures has not been widely evaluated.^5–9^ Although there were some studies focusing on QoL after breast and craniofacial reconstruction, there were no studies assessing these aspects in lower limb reconstruction.^10,11^ Along with innovative surgical procedures of lower extremity reconstruction, there has been a growing interest not only to evaluate functional outcomes but also to assess QoL.^12^ QoL was measured with questionnaires, with several different questionnaires available. The advantage of this evaluation lies in its easy use in clinical practice, and therefore, such questionnaires have acquired wide acceptance after extensive modifications and field trials. The aim of the study was to compare QoL after lower extremity reconstruction with pedicled and free flaps. Only very little was known about the QoL after limb reconstruction with these techniques. The hypothesis was that more complex means of reconstruction will lead to better clinical results and thus to higher QoL.

## MATERIAL AND METHODS

**Fig. 1.**
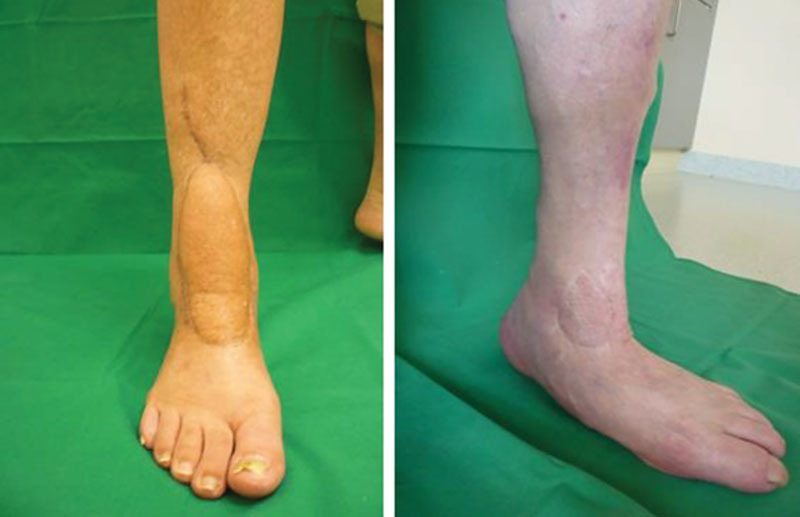
Reconstruction after ALT flap (A) and suralis flap (B).

The distally based adipofascial sural flap [pedicled reverse sural flap (PRSF)]^13^ and the free anterior lateral thigh (ALT) flap^14^ were established solutions for posttraumatic lower limb defects. Both flaps were workhorses in the institution where the study was conducted, resulting in wound healing rates of 95% in both flap types (Fig. 1). A specific questionnaire was developed to measure the patient’s QoL after reconstructive surgery with a flap and described as Bavarian Plastic Surgery Questionnaire. It was assembled out of 4 well-established health care scores and a group of specially designed questions regarding the social and work life and the private behavior of patients. To gain valid and objective data for QoL in the cohort, a questionnaire addressing results after reconstructive procedures was developed in cooperation with the institute for Medical Psychology of the University of Wuerzburg. The questionnaire combined questions from established scores as the short form health survey-12,^15^ the Dresden Body Image Score-35,^16^ the Patient Health Questionnaire (PHQ)-4,^17^ a questionnaire focusing on joint function [X-SMFA],^18^ and additional questions on issues not addressed in the established questionnaires. Thirty-seven patients with reconstruction of lower limb defects treated with a sural flap and 34 patients treated with an ALT flap were included in the study.

The QoL was assessed retrospectively at the time of the questionnaire, with a minimum follow-up time of 9 months. Inclusion criteria were full thickness skin defect located in the distal third of the lower leg and foot, flap coverage with either an ALT or a PRSF, and a follow-up time of ≥9 months. Exclusion criteria were history of psychiatric illness, inappropriate knowledge of the questionnaire language, and seriously flawed questionnaires. Between 2009 and 2012, 55 patients treated with ALT flap and 77 patients with PRSF could be identified. After application of the exclusion criteria, 37 patients treated with PRSF and 34 patients with ALT flap were included in the study. Within the assessment of the questionnaire, a photograph documentation and physical examination were conducted. The following demographic data were compiled: sex, age, weight, height, occupation, and side of reconstruction. Complications were evaluated and included: skin flap necrosis, infection, reconstructive failure, and need for revision surgery. The study was approved by the institutional ethical committee, and written informed consent was obtained from each patient. A statistical power analysis was performed, and the minimal number of patients was set to be n = 32 (β > 0.8). Statistical analysis was performed using SPSS version 14 (IBM, Armonk, NY). Continuous variables were analyzed using *t* test, and categorical variables were analyzed using the Mann–Whitney *U* test. Differences were considered as statistically significant, when *P* < 0.05.

## RESULTS

**Fig. 2.**
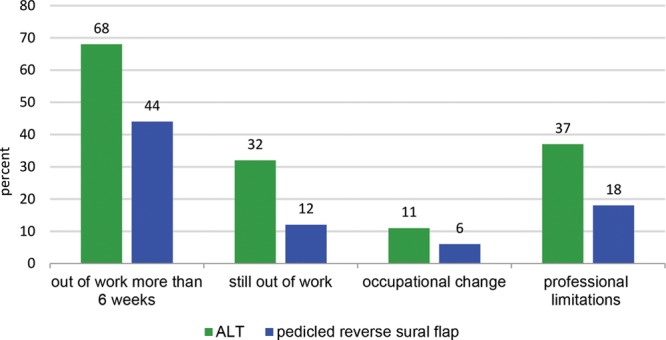
Occupational effects, *P* > 0.05, no significance.

**Fig. 3.**
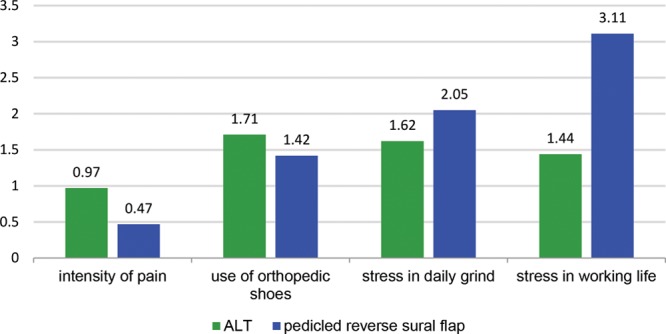
Limitation in daily life. 0: Never to 5: always.

Thirty-seven patients treated with a PRSF and 34 patients with an ALT flap were included with 85% of the patients being male in both groups, respectively. We performed a multivariant analysis of the types of wounds. Mean patient age was 57 years in the PRSF group and 44 years in the ALT group which displayed a significant difference; furthermore, 47% of the PRSF group patients were retired at the time of surgery compared with 21% of the ALT group. To prove the equality of both groups, we did a statistical analysis of the patients characteristics. We could show that there was no statistical significance concerning the type of wounds (posttraumatic, post-tumor resection, postinfectious, and chronic ulcers), the anatomic area (medial ankle, lateral ankle, distal third of the lower leg, foot), wound depth, comorbidities, presence of osteomyelitis, and the time of hospitalization. The mean size of the defects was 134 cm^2^ (±116 cm) in the ALT group and 42 cm^2^ (±24 cm) in the PRSF group which was a significant difference. The time of surgery was 86 minutes (±39 minutes) in the PRSF group and 248 minutes (±57 minutes) in the ALT group. The difference was significant. Indications for soft-tissue reconstruction were primary soft-tissue defect after trauma (52% ALT, 39% PRSF), impaired wound healing leading to subsequent for flap coverage (37% versus 44%), infection (5% versus 17%), and tumor (6% versus 0%); the difference between both groups was not significant. Twenty percentage of the patients in the PRSF group suffered a partial necrosis of the flap, which did not require revision surgery and healed with conservative treatment. Within the ALT group, 1 total flap necrosis was observed which had to be addressed with another free flap (ALT of the injured extremity). Secondary flap thinning was performed in 26% of the patients in the ALT group, whereas no secondary procedures were necessary in the other group. There was no significant difference in the postoperative hospital stay, but patients treated with ALT flap had a longer return to work period and needed to change their work environment more frequently (Figs. 2 and 3).

**Fig. 4.**
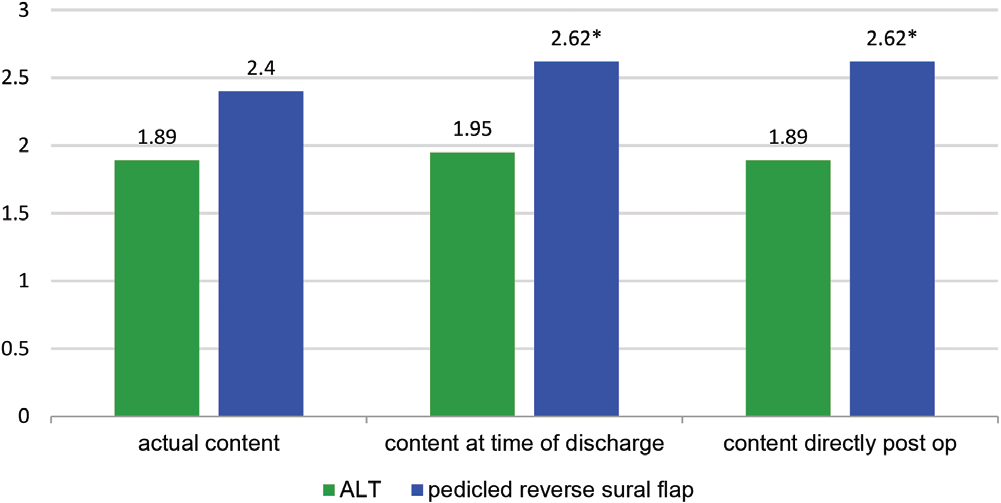
Content with surgical treatment. 0: Very satisfied to 5: very unsatisfied.

There was no statistical significant difference in the overall satisfaction with the procedure in the long-term follow-up between both groups, but patients with ALT showed a higher satisfaction with the treatment in the initial postoperative period (Fig. 4).

**Fig. 5.**
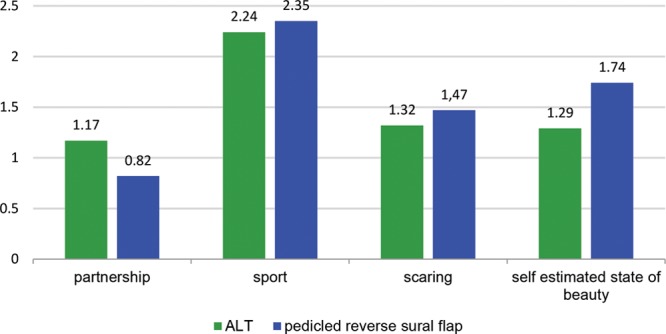
Limitation in private life. 0: Not existing to 5: very strong.

The stress induced by the procedure was rated by the short form health survey-12 score^15^ as medium without significant differences between both groups. Patients treated with a PRSF flap showed a significantly better overall physical state in the initial postoperative time, but these differences could not be detected in the long-term follow-up. Patients with a PRSF flap more often needed special orthopedic shoe equipment. On the other hand, ALT patients felt more affected by their flap in work life as well as daily private life. These differences failed to display statistical significance. Regarding spare time activities, 37% of the ALT group stated that they were unable to realize planned holidays, whereas only 14% of the PRSF group approved this statement. Twenty-five percent of the PRSF group felt ashamed to show the undressed operative limb in public (eg, swimming pool); this applied to 37% for the patients in the ALT group. No statistical significant difference was found for other limitations regarding public life. There was only a slight trend in the ALT group for limitations in fictive dating of a new partner because there were no significant limitations in existing relationships in both groups. There were no statistical significant differences in the groups in regard to overall sense of beauty and reduction of QoL through scarring formation. Questions regarding psychological impairment also showed no significant difference in both treatment groups (Fig. 5).

**Fig. 6.**
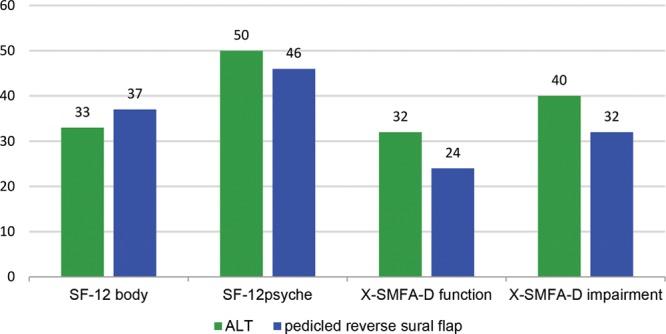
SF-12 (heath status), X-MFA (joint function) max. 100. SF indicates short form health survey.

The joint function (X-SMFA)^18^ is also highly influenced by the trauma to the joint itself, patient age, and preoperative degenerative joint changes. In the long term, PRSF patients estimated their joint function after flap reconstruction better than ALT patients. The preoperative data show the opposite. The same effect can be noted if the impairment of life quality caused by joint function is measured. In the long-term survey, the PRSF patients show a minor impairment of their lives caused by the injured joints. All the differences lacked of statistical significance (Fig. 6).

The Dresden Body Image Score-35 score^16^ displays the patient’s body image. The higher the score the better the QoL concerning the patients’ body perception. Both groups demonstrated approximately similar results in the long term for self-acceptance and vitality. Patients of the ALT group had a more positive body image before the trauma/surgery than patients in the PRSF group. Self assessment of sexual attractiveness showed a slight decrease in the long term for both groups without any significant difference. Body perception was not influenced in the long term.

**Fig. 7.**
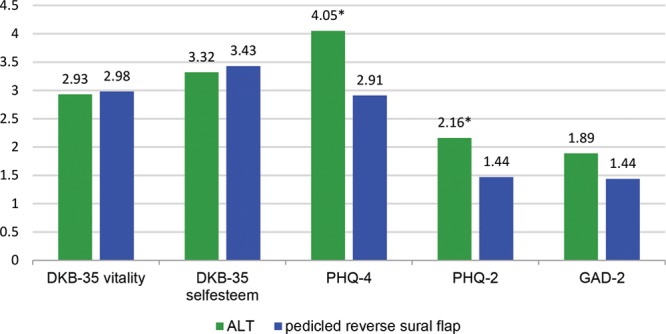
PHQ-4 score (depression and anxiety disorders); DKB-35 (body image). Maximum score: 5 (DKB); 12 (PHQ). DKB-35 indicates Dresden Body Image Score.

Psychological disorders were assessed using the PHQ-4 and PHQ-2 scores, with a focus on depression and anxiety disorders.^17^ Patients in the ALT group felt depressive mood changes more frequently and were modestly more anxious than PRSF patients in the long term, whereas these differences could not be observed preoperatively (Fig. 7).

## DISCUSSION

The convincing clinical results of free perforator flaps with minor donor site morbidity^19^ have largely expanded the variability of procedures addressing soft-tissue defects.^20^ Especially, the ALT flap has become a workhorse flap due to the reliable anatomy and its versatile use in soft-tissue reconstruction.^5^ Especially, in the lower extremity, local flaps and pedicled flaps could not be regarded as reliable due to the anatomical situation. In this region, the perforator/angiosome concept has also enabled surgeons to use pedicled flaps such as propeller perforator flaps or the distally based suralis flap (PRSF) with encouraging results.^6,13^ Although free perforator flaps require special infrastructure and environment and also resources, pedicled flaps do not require microanastomosis and therefore use lesser resources. Although the view of the microsurgeon nowadays favors free tissue transfer, the patient's opinion has largely remained unevaluated. The basic questions for microsurgeons may also be whether patients value the great effort of free flaps more than the “smaller effort” of pedicled solutions. Surgical results in this study are comparable to available literature.^21^ Partial flap loss in PRSFs is a documented sequela and cannot be avoided altogether.^22^ In contrast, free flaps will either show complete survival or loss of the flap, furthermore, more often necessitating secondary procedures, such as consecutive flap thinning. The surgical view regards a free perforator flap as a better and more durable choice than a pedicled flap. This view is not supported in patient’s estimation. General satisfaction with the procedure did not display statistical significant differences between both groups. Although satisfaction was higher in the immediate postoperative period after ALT flaps, this effect faded over time. The increased satisfaction in free flaps may be due to very frequent flap monitoring as opposed to pedicled flaps and comparatively higher attentiveness from surgeon and personnel. On the other hand, self-perceived, overall postoperative physical state of patients with PRSF was significantly better, not resulting in higher satisfaction. This may be attributed to the fact that trauma was less severe in this group (ie, also chronic wounds included), also not resulting in “exaggerated” satisfaction with “solution” of the problematic situation. Consecutively, patients in this group felt less affected in life afterwards, although requiring orthopedic footwear more often than ALT patients, and were less ashamed to present their reconstructed extremity in public. We could show 2 fairly comparable groups as to type of wounds, anatomic location, wound depth, comorbidities, presence of osteomyelitis, and time of hospitalization. Nevertheless, an explanation and thus limitation of this study is that within the small observed cohort, confounding variables such as the concomitant bone and joint trauma, age, pretraumatic degenerative changes, and different occupational status were not distributed equally. The difference in age distribution in both groups demonstrates this. Whereas nearly half of the PRSF patients were retired at the time of the surgery, only 21% of the ALT patients were retired. This inevitably results in a decrease of occupational limitations in the PRSF group. Therefore, the conclusion that the reconstruction method with a PRSF has an effect on occupational aspects must not be drawn because an unbiased comparison is impossible. Although attempts to avoid the influence of the initial trauma, infection and tumor were made by focusing the additional questionnaire (Bavarian Plastic Surgery Questionnaire) only on the functional aspect of flap reconstruction, it remains a limitation of this observational study that the outcome may be caused by feasibility of surgical options rather than by treatment choice alone. On the other hand, surgeon’s and patient’s decisions are influenced by prognostic and anatomical factors, which can never be ruled out altogether.

Although anatomic situation may dictate flap choice coverage with free flaps, a less-complicated flap is by no means regarded as an inferior treatment option in patient’s estimation. Patients value stable closure most, which should encourage surgeons also to offer other solutions than free flaps whenever possible. Although the value of flap techniques is often regarded based on the required skills in the surgical community, patients value solely the result devoid of the technical challenge. Despite the intuitive speculation that patients with more advanced reconstruction methods should have better function and subsequently higher QoL, this assumption was clearly not supported by data in this study. Although expected QoL cannot overrule objective criteria such as defect size, demand for tissue quality etc., it may be the most important aspect for patients in the long term and thus has to be taken into consideration. Although data in breast reconstruction may suggest that the most complex technique of reconstruction (autologous-free breast reconstruction) yields superior results in terms of longevity, this conclusion may not be applicable to lower extremity reconstruction.
